# Associations Between Regional Brain Volumes and Dual Decline in Gait Speed and Memory

**DOI:** 10.1002/alz70856_101761

**Published:** 2025-12-24

**Authors:** Sadhani Karunarathna, Monique Breslin, Jane E. Alty, James Scott McDonald, Richard Beare, Velandai K Srikanth, Taya A Collyer, Michele L Callisaya

**Affiliations:** ^1^ Menzies Institute for Medical Research, University of Tasmania, Hobart, TAS, Australia; ^2^ Royal Hobart Hospital, Hobart, TAS, Australia; ^3^ Wicking Dementia Research and Education Centre, University of Tasmania, Hobart, TAS, Australia; ^4^ School of Psychology, Newcastle University, Newcastle upon Tyne, Tyne and Wear, United Kingdom; ^5^ National Centre for Healthy Ageing, Melbourne, VIC, Australia; ^6^ Murdoch Children's Research Institute, Royal Children's Hospital, Melbourne, VIC, Australia; ^7^ Peninsula Clinical School, Central Clinical School, Monash University, Melbourne, VIC, Australia; ^8^ Frankston Hospital, Peninsula Health, Melbourne, VIC, Australia

## Abstract

**Background:**

Dual decline in gait and cognition is associated with an increased risk of dementia, with the strongest association seen between gait speed and delayed memory. However, the underlying brain correlates remain unknown. This study aimed to explore the associations between regional brain volumes and dual decline in gait speed and delayed memory.

**Method:**

Participants over 60 years were randomly selected from the Southern Tasmanian electoral roll (Australia). Baseline brain MRI and three serial gait speed and delayed memory assessments were performed on average 2.5 years apart. Participants were classified into four groups depending on tertiles of annual decline in gait speed and memory: non‐decline, gait only, cognition only, and dual decline. Twenty‐one regional brain volumes (in frontal, parietal, temporal, subcortical, brain stem and cerebellar areas) were preselected based on previous studies of gait and memory. Multinomial logistic regression was used to examine the associations between baseline regional brain volumes and the four groups.

**Result:**

The mean age of participants was 70.9 ± SD 6.7 years (*n* = 266). Lower volume in six brain regions (superior frontal gyrus, anterior cingulate cortex, middle frontal gyrus, thalamus, orbitofrontal cortex, hippocampus) were associated with a higher risk of dual decline. Lower volumes in the thalamus and cerebellum were associated with a higher risk of gait only and cognitive only decline respectively. However, these associations did not remain significant after correction for multiple comparisons.

**Conclusion:**

In this exploratory study regions related to memory, executive function, motor, and sensory motor integration were found to have associations with dual decline. Larger studies investigating a wider range of brain pathologies are required to fully understand the mechanisms underlying dual decline.

